# Psychedelic-assisted psychotherapy: The need to monitor adverse events

**DOI:** 10.1177/02698811251340929

**Published:** 2025-05-21

**Authors:** KRJ Schruers, NK Leibold

**Affiliations:** 1 Department of Psychiatry and Neuropsychology, Mental Health and Neuroscience Research Institute, Maastricht University, Maastricht, The Netherlands; 2 Department of Health Psychology, University of Leuven, Leuven, Belgium; 3 Mondriaan Mental Health Center, Maastricht, The Netherlands

**Keywords:** Psychedelic-assisted therapy, adverse events, monitoring

## Abstract

The therapeutic use of psychedelics for mental health issues holds considerable promise. However, systematic assessment of adverse events associated with these substances has received relatively little attention. Here, we discuss several considerations concerning the assessment of adverse events in psychedelic-assisted therapies. We discuss the preference for using the term “adverse effects” over “side effects”, as well as the ongoing debate regarding which substances are classified as psychedelic. We also provide recommendations on when and how to assess adverse effects, for example the importance to study them in any kind of therapy involving psychedelics, and using comprehensive monitoring of a wide range of physical parameters in combination with behavioral outcomes and the individual’s experience, at baseline and throughout the study. Also, sex-specific differences should be considered. Furthermore, we highlight several significant studies that have addressed these aspects. In summary, psychedelics offer great promise as a potential treatment (add-on) option in psychiatry, but more rigorous assessment of adverse effects is needed to promote safe use and implementation in clinical practice.

Psychedelics offer a promising new avenue and therapeutic prospect in psychiatry, particularly after a long period with scarcely any real breakthroughs or novelties. In fact, most medications currently in use are based on derivatives of drugs that were discovered by chance in the 1950s (Nutt et al., 2020). The use of psychedelics has a long history in many parts of the world, but the increasing recreational use led to legal restrictions, which also discontinued research efforts for several decades. Therefore, psychedelics are only recently beginning to re-enter the field of psychiatric science, including formal tests of efficacy in randomized controlled trials (Rucker et al., 2018). However, in spite of accruing knowledge on potential side effects, relatively little attention has been devoted to systematically studying those (Breeksema et al., 2022), which is associated with very inconsistent reports regarding the occurrence and intensity of any adverse effects (Breeksema et al., 2022). The proposed assessment framework by Palitsky et al. (2024) is a timely call to action in that respect.

Palitsky et al. (2024) recommend that different assessors use appropriate (validated) measurement instruments at various time points and frequencies. While this is a well-considered approach, some issues deserve further consideration when preparing to study adverse effects in psychotherapies that are accompanied by a pharmacological compound such as psychedelics.

First of all, there is the choice of wording. In pharmacology, the term “side effects” is often used. “Side effects” are defined as any unintended or undesired effects of a pharmaceutical product occurring at a normal or therapeutic dose (Lappas and Lappas, 2022; World Health Organization, 2002). This term assumes a causal relationship between the treatment and the event at hand. However, in this relatively early state of affairs of studying psychedelics, with many yet to be explored aspects, keeping an open mind is of the essence. Therefore, the term “adverse events” is preferable, as it applies to all events that occur to the participant during the treatment, without necessarily implying a causal relationship (World Health Organization, 2002).

Second, similarly to the absence of a clear consensus on which term should be used to describe adverse effects, there is an ongoing debate on what substance should be regarded as a psychedelic. Psilocybin and lysergic acid diethylamide (LSD) are, for example, universally regarded as psychedelics, while 3,4-methylenedioxymethamphetamine (MDMA) is excluded from this category by some (Dunlap et al., 2018). This discrepancy stems from the different factors taken into account for categorizing the substances. Classic psychedelics alter someone’s mood, sensory perception, and perception of reality, and therefore someone’s state of consciousness (Preller and Vollenweider, 2018). MDMA changes someone’s mood and sensations, but does not have the same intense effects, such as, for example, vivid (visual) hallucinations that are typically associated with psychedelics (Dunlap et al., 2018). In addition, also the mechanism underlying their effects are different: classical psychedelics exert their effects mainly through activation of the serotonin 2 A receptor (Nutt et al., 2020; Preller et al., 2018; Vollenweider et al., 1998), while MDMA acts more on inhibiting reuptake transporters and therefore mainly acts as an enhancer of extracellular levels of monoamines (Green et al., 2003; Dunlap et al., 2018). Despite these differences in induced phenotypes and molecular mechanisms, in the current state of affairs, it seems advisable not to be too restrictive in the list of compounds to which the term psychedelics applies.

Third, assessing adverse effects in psychotherapies does not receive enough attention. Pharmacological-assisted psychotherapy assumes at least two parts of treatment: a drug and some form of psychological therapy with a set of instructions, formally structured to different degrees, depending on the type of therapy at hand. The essence of using psychopharmacology in this context is to induce changes in the brain and mind of patients using chemical substances to increase the effect of the given psychotherapy (Lochtenberg et al., 2021). In this respect, using psychedelics as compound in addition to a therapy is not different at all. As other pharmacological compounds, psychedelics are used to alter someone’s perception and experience, expecting that this could boost the efficacy of a treatment. In studies in which a pharmacological compound is administered to participants, particularly in clinical trials, it is common practice to assess and report any adverse events to determine the safety of the given drug and dose. Therefore, comprehensively assessing adverse effects of psychotropic drugs is of high relevance as well.

Interestingly, even when psychological therapies are used without any additional compound, they are sometimes assumed not to be associated with adverse events at all. In recent years, however, it has become increasingly clear that this is not the case and formal research into this subject is slowly but gradually increasing (Holze et al., 2024; Klatte et al., 2023). In addition, the lack of a uniform consensus on what “psychotherapy” entails adds to the challenge of when to monitor adverse effects. The set of actions used varies vastly between institutes and clinicians, and different opinions exist on whether or not the interpersonal support that is given during psychedelic-assisted therapy (PAT) is to be labeled as psychotherapy. At the very minimum, information on the effects of the drug is provided to the participant, along with general instructions on how to behave. These instructions often include taking a relaxed reclined position, keeping an open mind and being invited to share any experience with the professionals that are present (Mithoefer, 2017). Often, this “psychotherapeutic” part in itself is not supported by any evidence of effect, even in highly influential studies (Mitchell et al., 2021, 2023). Therefore, adverse effects should be assessed in a broad sense in any kind of therapy.

## What should be assessed in PAT?

Obviously, a broad range of physical signs should be closely monitored as long as psychedelics are not yet approved for and implemented into daily clinical practice. Physical parameters should include vital signs such as blood pressure and heart rate but also blood analysis should be taken into account to monitor parameters relevant for the specific compound that is used in combination with the therapy. For these physical parameters no need for tailoring exists, as the standard practice of medication trials can be applied in PAT as well.

Several significant studies have been done that address physical parameters (and subjective experiences), but are not discussed by Palitsky et al. (2024). For example, well-designed double-blind, randomized, placebo-controlled studies demonstrated that psychedelics such as LSD (Schmid et al., 2015) and MDMA (Vizeli and Liechti, 2017) elevate blood pressure and heart rate. These studies also provided a comprehensive assessment of other parameters, revealing that psychedelics increase body temperature (Schmid et al., 2015; Vizeli and Liechti, 2017), as well as pupil size after light stimulation and in the dark, levels of plasma cortisol, prolactin, oxytocin, and epinephrine (Schmid et al., 2015). Moreover, while MDMA did not affect kidney and liver function 4 weeks post-administration, it led to a decrease in red blood cell counts and hemoglobin levels (Vizeli and Liechti, 2017), showing the broad effects psychedelics can have on the body. Interestingly, these effects on red blood cells and hemoglobin were observed in women only, emphasizing the need to consider sex-specific differences in psychedelic research.

In addition to monitoring physical parameters, it is also essential to thoroughly assess an individual’s experience. Under supervised administration in research settings, psychedelics are generally well tolerated and produce positive effects (Holze et al., 2019, 2022; Schmid et al., 2015). For example, both short- and long-term improvements in well-being have been observed (Schmid and Liechti, 2018), and many studies report improved mental health, including a decrease in suicidality. More specifically, suicidal ideation decreased within 8 h after psilocybin administration, with effects persisting up to the 6.5-month follow-up (Ross et al., 2021). A similar reduction in suicidality was reported within less than an hour after administration of ayahuasca, with lasting effects at the 21-day follow-up (Zeifman et al., 2021), and for psilocybin that decreased suicidality scores for 2 weeks (Carhart-Harris et al., 2018). In the latter study, the decreased suicidality scores did not remain statistically significant at the 5-week follow-up. A recent meta-analysis of seven studies confirmed that psychedelics acutely reduce suicidality, with effects lasting for up to several months (Zeifman et al., 2022a, 2022b). However, in some cases, an increase in suicidality has been observed (Goodwin et al., 2022; Zeifman et al., 2022a, 2022b), and some individuals experience “bad drug effects” (Holze et al., 2019, 2022; Schmid et al., 2015; Vizeli and Liechti, 2017) or anxiety (Holze et al., 2022) after taking a psychedelic. In some instances, serious adverse events have also been documented, including acute anxiety and delusions requiring pharmacological intervention (Holze et al., 2023). It remains unclear which individuals are most susceptible to negative or adverse effects, but in line with the previously mentioned sex-specific differences in psychedelic-induced physical responses, women may be more prone to acute adverse effects, such as lack of appetite and anxiety, within the first few hours after administration and up to 24 h afterward (Vizeli and Liechti, 2017). These sex-related differences persist even when the psychedelic dose is adjusted for body weight (Liechti et al., 2001; Vizeli and Liechti, 2017).

As outlined in the framework proposed by Palitsky et al. (2024), researchers in part rely on participants’ self-reports to evaluate these subjective experiences. Since PATs are distinctive in terms of the phenomenology they induce, including “trips,” visual or perceptual distortions, psychological insights, mystical-type experiences (Pedersen et al., 2021), and the impact on an individual, it has been argued that a more tailored approach is necessary to assess adverse events. This perspective led to the assessment model proposed by Palitsky et al. (2024). However, a suitable framework already exists in the form of the standard Mental State Examination, covering (changes in) consciousness, attention and concentration, memory, appearance, behavior, mood and affect, thought process and content (including delusions), perception (including illusions and hallucinations), and insight (Soltan and Girguis, 2017). This examination thus covers a broad range of aspects. The matter may be different regarding the sometimes occurring longer-lasting effects on participants’ meaning systems, world views, and spiritual or existential outlooks (Palitsky et al., 2024). These are not commonly observed in psychopharmacological practice but can be a result of psychotherapy and are therefore also relevant to assess. However, as opposed to some observations in PAT, such changes are only to be expected after a prolonged time in psychotherapy.

## How should these assessments be conducted?

Physical parameters should be assessed comprehensively. Vital signs such as blood pressure should be measured continuously to allow accurate monitoring of changes with a high temporal resolution. With regard to behavioral effects and an individual’s experience, these are classically assessed using standardized questionnaires, either self-administered or via interview, in the framework of pharmacological studies. In PAT, ideally, such an assessment instrument should be complete (i.e., covering all above-mentioned aspects), in lay terms to make it understandable for participants, quick to administer, possessing good psychometric properties (internal and external validity, reliability, and norming), and allowing for quantitative comparisons. While it is desirable that assessments are complete, it is debatable which items should be part of the assessment to make it “complete.” To design a standardized measurement instrument with good psychometric properties, it is important to avoid bias in the selection of the items to be included. For this purpose, it is advisable that the development of such an instrument starts with a qualitative phase. Current qualitative research methods consist of rigorously conducted interviews with stakeholders, crucially including participants and their environment (Moser and Korstjens, 2018). In an individual or a group setting, input is sought in an open, unbiased fashion until no new analytical information arises (i.e., when data saturation is reached). Next, labels are made based on the data and clustered into categories or themes to gain a deep understanding of participants’ perceptions (Moser and Korstjens, 2018). This information can form a solid basis for important aspects that should be included in a quantitative scale.

## When should these assessments take place?

Ideally, as already proposed by Palitsky et al. (2024), assessments should take place repeatedly throughout the study period. Before starting the procedure, a baseline should be established. This baseline should take into account attitudes and expectations toward psychedelics as these can have a considerable impact on (placebo) effects (Aday et al., 2022). During the procedure, the effect on an individual and his/her experience should be closely monitored. As it has been previously shown that participants’ reports during the procedure might not be reliable (Carhart-Harris et al., 2015), it seems wise to complement these reports with an interview of an observer (preferably independent) and the attending clinician.

Often, monitoring any adverse effects ends when a participant leaves the research institute, because it is believed that the impact has worn off sufficiently to send the participant home safely. However, PATs can also lead to longer-lasting effects, for example, on meanings and beliefs, self-knowledge, and individuals’ daily behaviors and emotions (Palitsky et al., 2024). LSD has been reported to have subjective positive effects on well-being even after 1 year (Schmid and Liechti, 2018). While adverse effects seem to be transient (Schmid and Liechti, 2018; Schmid et al., 2015), follow-up measurements after a period of several months are advised to capture these longer-lasting effects, possibly by making use of momentary assessment technology via a smartphone app, of which several are available.

## Conclusion

In summary, the re-emergence of psychedelics as a potential treatment (add-on) option in psychiatry offers both considerable promise and challenges. While there is increasing research on evaluating the potential benefits in the treatment of mental health issues, the assessment of adverse effects is inconsistent and has received little attention to date. In the future, a uniform terminology, comprehensive and repeated monitoring of physical and self-experienced effects, and long-term follow-ups are warranted. Also, sex-specific differences should be considered. This approach will contribute to establishing a safe use and potentially the implementation of psychedelics into clinical practice.
